# Scaling Law for Kasha’s Rule in Photoexcited Molecular Aggregates

**DOI:** 10.1021/acs.jpca.4c00342

**Published:** 2024-04-03

**Authors:** Raphael Holzinger, Nico S. Bassler, Helmut Ritsch, Claudiu Genes

**Affiliations:** † Institute for Theoretical Physics, Innsbruck University, Technikerstraße 21a, 6020 Innsbruck, Austria; ‡ Max Planck Institute for the Science of Light, Staudtstraße 2, D-91058 Erlangen, Germany

## Abstract

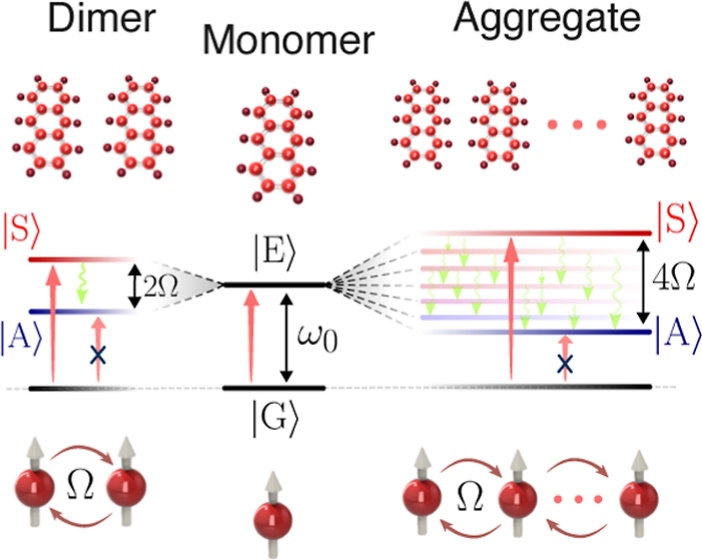

We study the photophysics of molecular aggregates from a quantum optics perspective, with emphasis on deriving scaling laws for the fast nonradiative relaxation of collective electronic excitations, referred to as Kasha’s rule. Aggregates exhibit an energetically broad manifold of collective states with delocalized electronic excitations originating from near-field dipole–dipole exchanges between neighboring monomers. Photoexcitation at optical wavelengths, much larger than the monomer–monomer average separation, addresses almost exclusively symmetric collective states, which for an arrangement known as H-aggregate show an upward hypsochromic shift. The extremely fast subsequent nonradiative relaxation via intramolecular vibrational modes populates lower energy, subradiant states, resulting in effective inhibition of fluorescence. Our analytical treatment allows for the derivation of an approximate scaling law of this relaxation process, linear in the number of available low-energy vibrational modes and directly proportional to the dipole–dipole interaction strength between neighboring monomers.

## Introduction

Molecular aggregates^1−3^ are self-ordered arrangements of monomers showing strong collective optical transition dipole strengths. Owing to the dense packing of monomers, with monomer–monomer separations at the order of a nanometer and total aggregate lengths of tens or hundreds of nanometers, thus much below an optical wavelength and despite inhomogeneous broadening and separation disorder, they exhibit delocalized excitons.^4^ This allows strong coupling to external light modes, resulting in collectively modified fluorescence rates. Following the discovery of J- and H-aggregates in the 1930s by Scheibe et al.^5^ and Jelley,^6^ their standard understanding is based on the original approach introduced by Kasha in the 1960s.^7^ Currently, J-aggregates are widely employed in light-matter coupling experiments aiming at the modification of material properties via the manipulation of the electromagnetic vacuum mode density around electronic resonances.^8^ Numerous approaches to understand their aggregation behavior and the subsequent response to external illumination have been taken, as thoroughly reviewed in ref (1).

**Figure 1:**
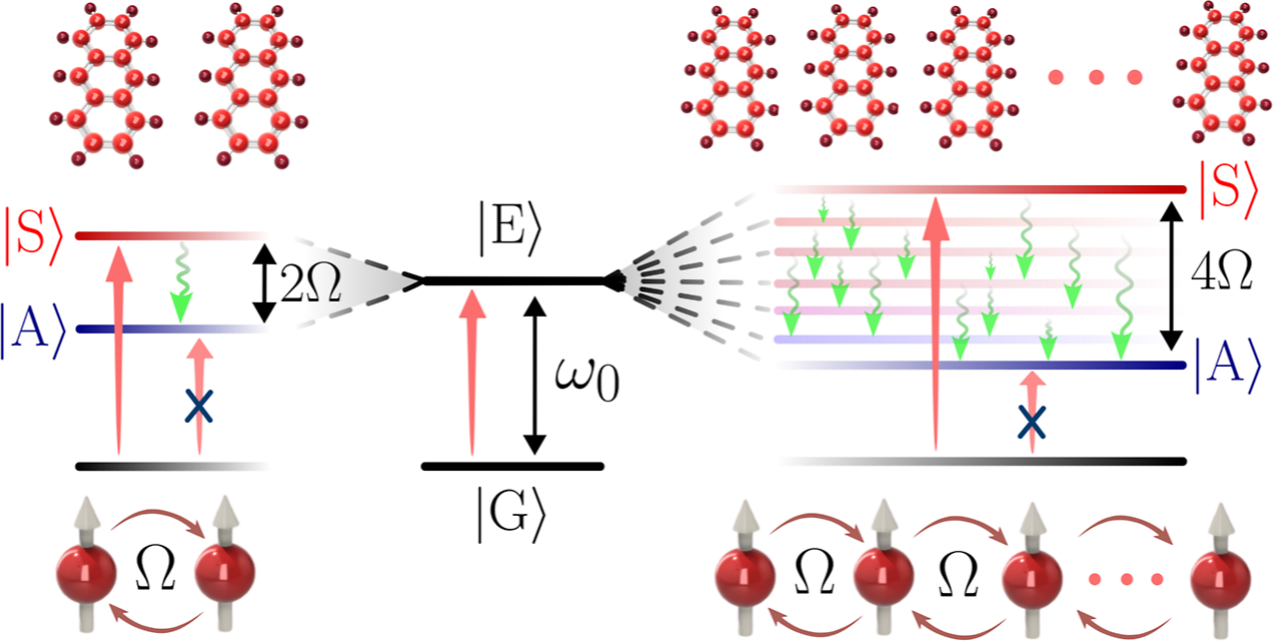
Illustration of the Kasha process in an H-aggregate for a dimer (left) and larger aggregate (right). The population migrates from the optically addressed state |*S*⟩ (superradiant, therefore color-coded in red) toward the lower and subradiant collective states |*A*⟩ (color-coded toward blue) via a multitude of vibrational relaxation channels (indicated as green arrows). The energy splitting in the large aggregate is four times the nearest neighbor dipole–dipole coupling strength Ω and strongly depends on the aggregate separation at the level of nanometers. A resulting effective relaxation rate from the high energy optically addressed state to the bottom of the energy manifold results, on the femtosecond time scale.

There has been recently a new surge in interest in theoretically understanding the behavior of organic molecular systems under the action of confined vacuum fields, i.e., within the context of cavity quantum electrodynamics.^9−12^ Among many approaches taken, a purely quantum optics formalism has been introduced, which is based on a simple model for electron–vibron interactions contained within the formalism of the Holstein Hamiltonian and which can incorporate loss of photons or vibrations via an open system approach following either a master equation or quantum Langevin equation approaches.^13,14^ Within such a quantum optics approach, rate equations for the dynamics of a molecular aggregate upon photoexcitation with light at visible wavelengths λ can be derived. As pointed out in ref (1), the dynamics of the exciton in the aggregate can be understood in terms of bright (or symmetric) and dark (or asymmetric) states. In the simplest example, a molecular dimer in the side-by-side H-aggregate configuration (see Figure 1) exhibits a symmetric superposition  sitting at energy ω_0_ + Ω. Here |*eg*⟩ denotes that the first monomer is in the excited electronic state while the second one is in the ground state and Ω is the nearest neighbor dipole–dipole interaction scaling with the monomer separation as *d*^–3^. As *d* is typically much smaller than λ, the asymmetric state, at energy ω_0_ – Ω, is decoupled from external light and instead is reached indirectly from the symmetric state |*S*⟩ via redistribution of energy into molecular vibrations, consistent with the standard understanding of a Kasha process. This can be easily generalized to a mesoscopic-sized aggregate, where an additional large number of dark states fill the 4 Ω frequency split between the photoexcited symmetric state and the lowest energy state. The Kasha incoherent loss of energy is mediated by a multitude of vibrational relaxation pathways. For a molecule with  atoms, a large number of the order  vibrational modes are available; some of them, up to some index *n*_max_, find themselves within the interval 4 Ω, thus providing resonant transfer between the set of collective electronic states.

We show that an approach based on rate equations allows for an approximate expression of the rate of a Kasha process as
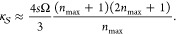
1The simplicity of the result lies in the small number of parameters involved: the average estimated Huang–Rhys factor , the monomer separation *d* (appearing via Ω), and the estimated number of intramolecular vibrations *n*_max_ of relevant frequencies. As an example, we consider the aqueous dye molecule Cresyl Violet (CV) which aggregates in an H-configuration, featuring more than 30 vibrational modes in the range ∼5–50 THz with a zero-phonon transition wavelength λ_0_ = 590 nm and a monomer fluorescence line width γ_0_ = 454 MHz.^15^ The nearest neighbor dipole–dipole coupling is estimated at around Ω = 23 THz and the vibrational spectrum is extracted from ref (16). We estimate around *n*_max_ = 12 vibrational modes to have a non-negligible Huang–Rhys factor larger than 0.01 and to fall into the range of the electronic energy band [0, 4 Ω]; this leads to an average estimated Huang–Rhys factor *s* = 0.09. The estimated time scale for the Kasha process is then around  fs. Similar estimates for Rhodamine 800^17^ and Bacteriochlorophyll a (BChla)^18^ give values for the Kasha process time scale of order 30 and 34 fs, respectively.

## Methods

**Figure 2:**
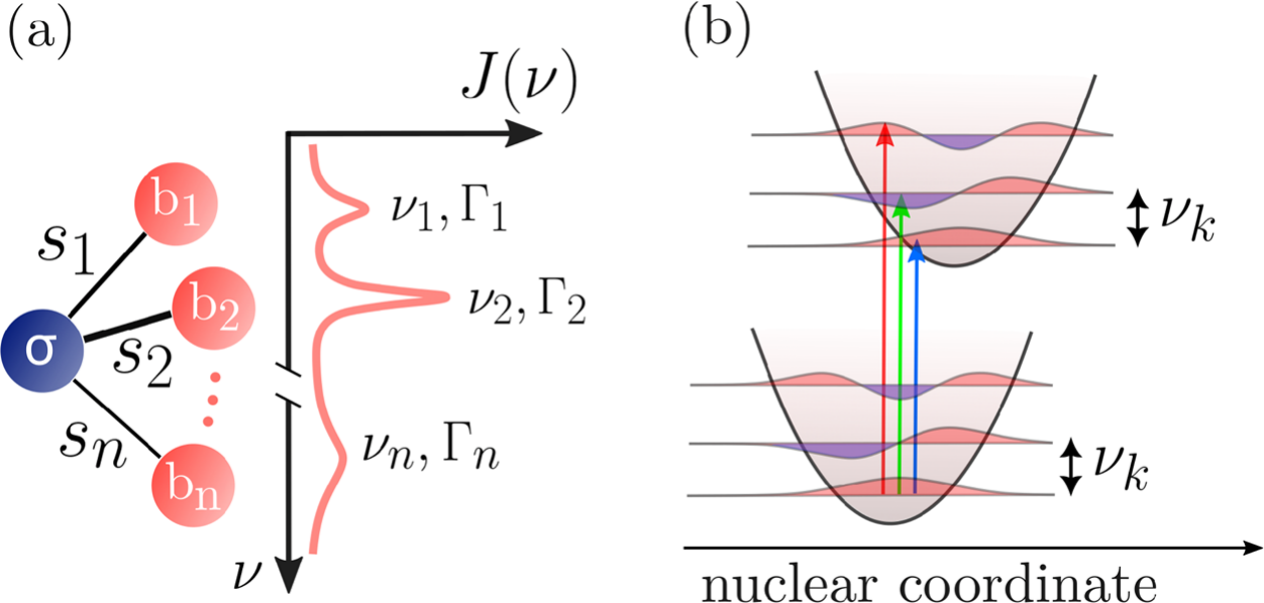
(a) Diagrammatic description of electron–vibron interactions within each monomer. A single optically addressable electronic transition operator σ is coupled to *n* vibrational mode operators *b*_*m*_ with frequencies ν_*m*_, line widths Γ_*m*_, and Huang–Rhys factors *s*_*m*_, where *m* runs from 1 to *n*. The vibrational spectral density *J*(ν) has a number of vibrational frequencies ν_*m*_ and associated relaxation rates Γ_*m*_ which dictate their line widths. (b) Ground and excited electronic potential energy landscapes along a single nuclear coordinate under the harmonic assumption. The vertical lines illustrate the Franck–Condon principle where substantial transition rates only are possible when a match between the symmetry of the nuclear wavepacket in the ground and excited electronic states.

The minimal model we consider involves a one-dimensional molecular chain of identical monomers with a typical intermonomer distance *d* in the nm range. As photoexcitation with wavelength 2π/*k*_0_ (with *k*_0_ the wavevector) is in the μm range, the condition *k*_0_*d* ≪ 1 is fulfilled, meaning that the chain is uniformly illuminated. Each monomer is assumed to undergo a single electronic transition, which in turn is coupled to *n* vibrational modes (see Figure 2). Each vibrational mode has a frequency ν_*m*_ and relaxation rate Γ_*m*_, where *m* runs from 1 to *n*. The monomer can undergo spontaneous emission at a rate γ_0_, owing to the coupling to the electromagnetic environment. For each monomer *j*, the electronic transition is at frequency splitting ω_0_ (*ℏ* = 1) and is described by the collapse operator σ_*j*_ = |*g*⟩_*j*_⟨*e*|_*j*_. The vibrational degrees of freedom are described by bosonic operators *b*_*jm*_ satisfying the commutation relations . The vibronic couplings are illustrated in Figure 2a as links between the electronic and vibration operators with magnitudes characterized by the Huang–Rhys factors *s*_*m*_. The electronic and vibrational degrees of freedom are subject to loss quantified by spontaneous emission rate γ_0_ and vibrational relaxation rates Γ_*m*_, respectively. The Hamiltonian for all  monomers is obtained as a sum over each particle’s Hamiltonian
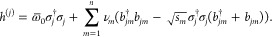
2Notice that the bare frequency is Stokes shifted . This shift will later be eliminated after a polaron transformation (see Supporting Information for more details). The model and its validity and relevance are extensively discussed in ref (13).

Dipole–dipole exchanges at rates Ω_*jj*′_ have a strong imprint at nm distances, owing to their scaling with the inverse cube of the particle separation  in the near-field region.^19^ This can be listed in the Hamiltonian as  and describes an excitation transfer between pairs of monomers via a virtual photon exchange. The coherent exchange is mediated by the dipole–dipole frequency shifts Ω_*jj*′_, which in units of the optical emission rate γ_0_ are given by , namely, proportional to the real part of the Green’s tensor in free space (shown in the Supporting Information). In the following, we will consider the particular case of side-by-side arrangement, where all transition dipoles  are parallel to each other and perpendicular to the chain direction.

**Figure 3:**
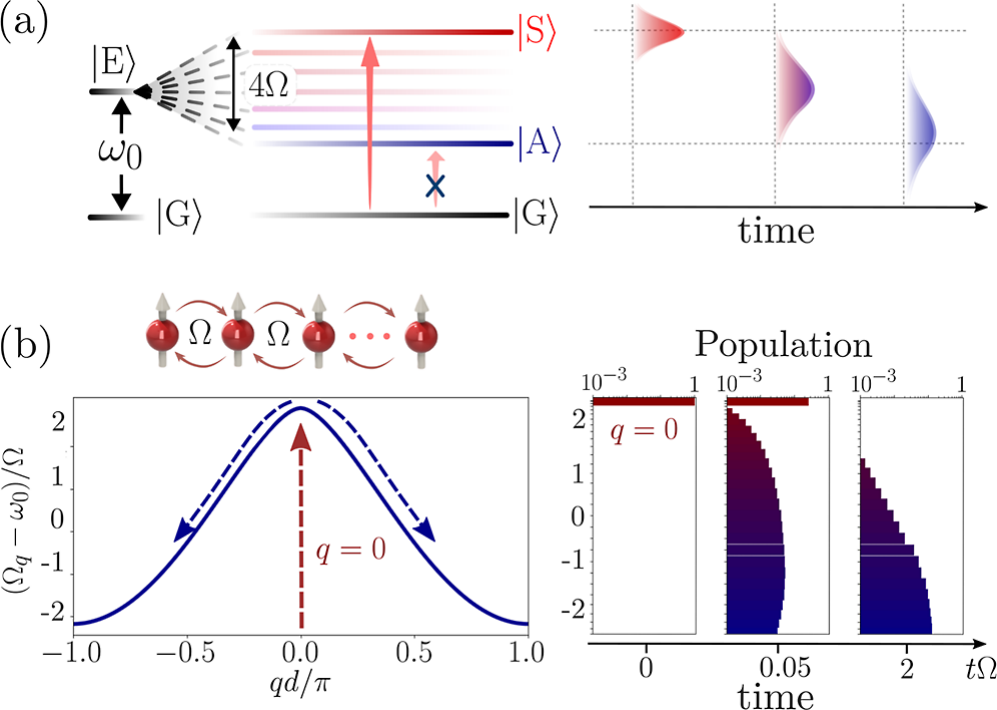
Dynamics of the collective state populations upon photoexcitation of the symmetric state in H-aggregates. (a) Illustration of the population migration in time from the optically excited state (superradiant and therefore color-coded in red) toward the lower and subradiant collective states (color-coded toward blue) via a multitude of vibrational relaxation channels. (b) Exact numerical time dynamics for a chain of  monomers. In such a mesoscopic limit, the analysis can be performed on a band diagram where the symmetric state, photoexcitable, sits at *q* = 0. The numerical simulation shows the time dynamics of populations on a time scale normalized to Ω^–1^.

The model can be better tackled on a collective basis^14^ resulting from the diagonalization of the first excitation subspace including dipole–dipole interactions. This can be done either in the discrete space where states are indexed from 1 to  (see Figure 3a) or alternatively, with the quasi-momentum index  obtained by a rescaling of the index of the mode  (see Figure 3b). This is based on the observation that, for aggregates with  of the order of a hundred monomers, the mesoscopic limit can be assumed such that the system can be considered translationally invariant and periodic boundary conditions can be invoked. A single symmetric mode can be distinguished with a state vector obtained by the application of the symmetric operator  to the collective ground state |*G*⟩. To a good approximation, the system can be considered to be in the Dicke limit where a single superradiant emission rate roughly estimated by  characterizes this ‘bright’ state. In addition, the other orthogonal  asymmetric states are obtained via the application of asymmetric operators  to |*G*⟩. These states are nonradiative at such deep subwavelength molecular separations, and we dub them therefore as ‘dark’.

The collective excitations are eigenstates of the dipole–dipole interaction Hamiltonian  where by definition we fix the symmetric shift . With periodic boundary conditions imposed, in the mesoscopic limit, one can derive the collective shifts as Ω_*q*_ = 2∑_*j*_Ω_1*j*_ cos(*q*(*j* – 1)*d*).^14,20^ Further simplifications occur by considering the nearest neighbor approximation and the collective eigenenergies become Ω_*q*_ = 2Ω cos(*qd*), where the nearest-neighbor coupling is simply denoted by Ω ≡Ω_12_. Closely following the procedure introduced in ref (14), one can analyze the coupling between states of different symmetries via electron–vibron couplings by an additional transformation to a collective basis for the vibrational degrees of freedom as well. This is done by introducing collective vibrational modes , with the momentum quadratures satisfying [*Q*_*q*_^(*m*)^,*P*_*q*_^(*m*)^] = *i* and *m* labels the vibrational mode running from 1 to *n*.

## Results

Owing to the small dimension of a molecular aggregate compared to an optical wavelength, under photoexcitation, only the symmetric collective mode is directly accessible. This immediately leads to a rescaling of the Rabi driving by a factor of : this can be seen equivalently as an increase of the oscillator strength by , thus rendering aggregates of any kind as good candidates for strong light-matter coupling. In addition, the particularity of the subsequent aggregate electronic dynamics lies within the shape of the energy band. For example, J-aggregates present an energy band where the symmetric mode lies at the bottom of the band, thus leading to a bathochromic frequency shift (to the left of the bare monomer frequency) and subsequently shows not only an enhanced absorption cross section but also enhanced fluorescence at a superradiant rate. In contrast, H-aggregates have a symmetric state located at the top of the energy band, corresponding to a hypsochromic shift. Most importantly, quick dynamics follows the optical excitation involving the relaxation of the collective state toward low-energy dark states. This dynamics is illustrated in Figure 3 in both the discrete and continuous cases. In Figure 3, a numerical simulation for  at an intermolecular spacing *k*_0_*d* = 0.0126. We have considered a large n spanning the range of frequencies from 0 to 4Ω, with Γ_*m*_ = ν_*m*_/10 and identical *s*_*m*_ = 0.01. Such time dynamics can be analytically tackled at the level of rate equations. We largely follow the derivation in ref (14) which we generalize here to incorporate the crucial aspect that many vibrational modes have to be taken into account. Under the assumption that the vibrational relaxation rates are fast compared to the coherent couplings and radiative loss rates, a set of rate equations for the populations of the symmetric state  and all dark states  can be derived (see Supporting Information). With the definitions —total loss rate of the symmetric state, κ_*q*_—loss rate for dark state *q*, —incoherent repopulation rate from the dark to the bright state and κ_*q*′→*q*_—incoherent rate for redistribution of energy within the dark state manifold, one can write
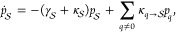
3a
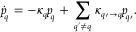
3bThe rate equations show that the symmetric state energy spills into the whole dark state manifold via rate  and, in addition, higher energy dark states spill into the lower energy ones via κ_*q*_. The quasi-unidirectionality of the process is ensured by the fact that, in this perturbative treatment, the coherent coupling between states is followed by quick vibrational relaxation, making the reverse process, governed by rates  and κ_*q*′→*q*_ from lower energy state to higher ones, very unlikely (as shown in ref (14) for the two monomer case). Mathematically, the condition is . Analytically, one can get an expression for the transfer rate between the symmetric mode and any dark mode *q* mediated by vibrational mode *m* as
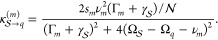
4and construct the total rate to all states spanned by the index *q* by summing  over all vibrations up to an index *n*_max_ within the frequency interval covered by 4Ω where the electronic collective states are positioned in energy. Moreover, we will consider the standard underdamped harmonic oscillator model for the molecular vibrations, i.e., the dissipation rate is much smaller than the resonance frequency for any mode Γ_*m*_ ≪ ν_*m*_. Equivalently, one can state that the quality factor of any vibrational mode is much larger than unity ν_*m*_/Γ_*m*_ ≫ 1.

**Figure 4:**
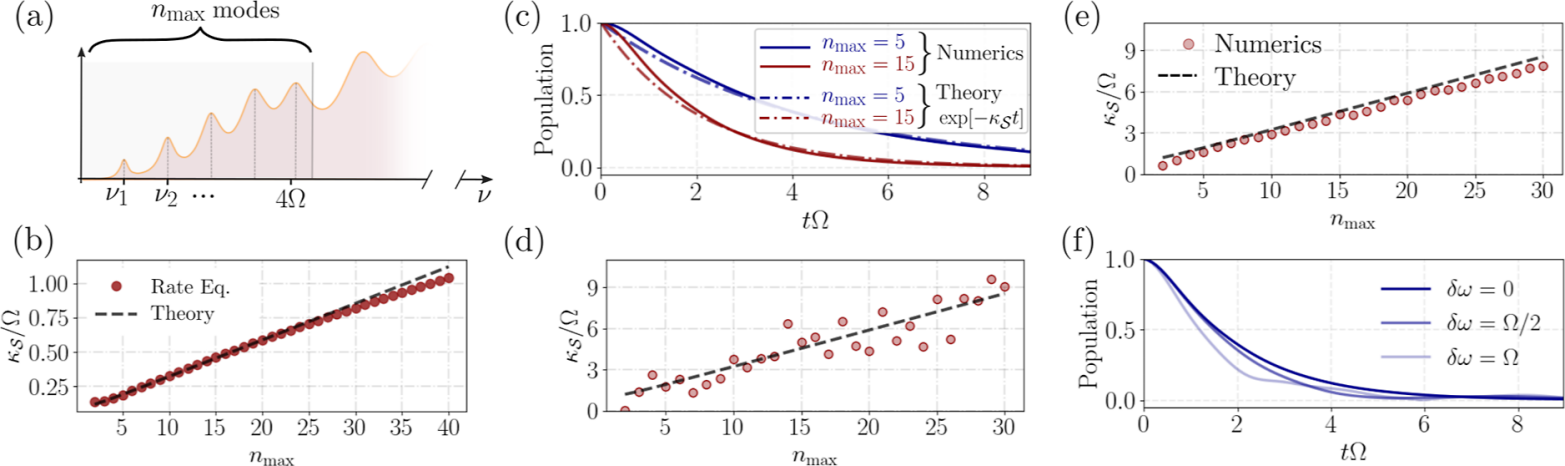
(a) Only a limited number of vibrational modes, from 1 to *n*_max_ can efficiently mediate resonant transfer between the symmetric state and the dark manifold. These modes are within the 4Ω bandwidth. (b) Linear scaling of the transfer rate  as a function of the number of vibrational modes, with the same parameters as in (c) and  molecules at *k*_0_*d* = 0.0126 separation. The scaling law from eq 5 provides a very good fit to the rate equations in eq 4. (c) Time dynamics of 20 molecules initialized in the symmetric state. Numerical results in the single excitation manifold show excellent agreement with an exponential decay given by , and governed by the analytical formula in eq 5 (Same parameters as in (d),(e)). (d) Numerical results for  plotted against increasing *n*_max_ for a randomly drawn set of vibrational frequencies ν_1_,···ν_*n_max_*_ in the window 0–4Ω and Huang–Rhys factors *s*_1_,···*s*_*n*_max__ in the interval [0,0.2]. The fit is performed with the analytical scaling in eq 5 for evenly spaced vibrational frequencies and Huang–Rhys factor at the level of the distribution average *s* = 0.1. (e) Further comparison of the analytical scaling from eq 5 with an average of over 200 random realizations shows almost perfect agreement. (f) Decay of the initial symmetric state population under the influence of random static frequency disorder with fluctuation δω around ω_0_. Further parameters in all plots:  molecules, *k*_0_*d* = 0.0126, Γ_*m*_ = ν_*m*_/10, Ω ≈ 3.759 × 10^5^γ_0_.

In order to further proceed with analytical estimates, let us first make some comments regarding typical time scales. Given that vibrational relaxation is on the order of tens to hundreds of GHz while spontaneous emission is in the range of tens of MHz, a very quick nonradiative path from the symmetric to low energy asymmetric states can be achieved on ps time scales. For monomer separations in the nanometer range, expected near-field shifts Ω in the range of 1 THz to tens of THz are expected. This means that only a few, low-energy, molecular vibrations can fit in the window of 4Ω (see Figure 4a) and aid the relaxation process. This allows us to derive an approximate scaling law for  as a function of a given number of vibrational modes *n*_max_ that can efficiently mediate the relaxation of the symmetric state into the dark state manifold. We proceed by first considering a given vibrational mode *m* and asking for the condition that this mode can transfer excitation from the symmetric mode to any of the dark states. We notice that with the condition that Γ_*m*_ ≪ ν_*m*_ (it is also implied that  even for large ) the resonance condition requires that the states to which resonant transfer can take place are only in the vicinity of the mode *q* fulfilling . Of course, in this case, one can immediately observe that any modes with ν_*m*_ > 4Ω cannot take part in this transfer. Assuming a constant density of all  collective states spread within the interval 4Ω, we can then estimate that a number of approximately  states fall close to the resonance , i.e., within the line width Γ_*m*_. Summing all of these contributions gives the total rate for all transitions mediated by mode *m* to states close to *q*. Summing over all possible relaxation paths that participate in the transfer giving thus an estimate for the total rate
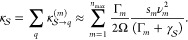
5In order to estimate this quantity, knowledge of the particular nature of the monomer’s frequencies, Huang–Rhys factors, and vibrational relaxation rates is necessary. However, while we will later numerically investigate random distributions of frequencies and Huang–Rhys factors, we aim first at deriving a simple scaling law. To this end, we will proceed by making some simplifying assumptions, among which the first is that the vibrational spectrum is equally spaced in the interval from 0 up to 4Ω. We denote the frequency of mode *m* by ν_*m*_ = *m*4Ω/*n*_max_. Let us also consider that all Huang–Rhys factors are equal to *s* (later we compare with a randomized distribution with an average *s*). Moreover, we neglect the contribution of  as it is much smaller than Γ_*m*_ (this should be typically very well fulfilled as γ_0_/Γ_*m*_ is expected to be around 10^–5^). Summing over all vibrational modes within the interval of 4Ω gives us an approximated scaling law as the expression already introduced in eq 1. The result shows the independence of the total number of monomers  and a quasi-linear dependence on the total number of available low-frequency vibrational modes which can resonantly participate in Kasha’s relaxation process from the high energy symmetric state to the bottom of the dark state manifold. Notice that the predicted time scale is dictated by the nearest neighbor dipole–dipole coupling strength Ω which in turn depends on the inverse cube of the monomer–monomer separation. In the first step, we can estimate that the analytical scaling is in very good agreement with the results of the rate equations, as shown in Figure 4b.

The validity of the analytically obtained result can be easily checked against the numerical simulations. In the first step, we compare the numerical results with the analytical scaling of eq 1, for an equidistant vibrational spectrum, in Figure 4c. A very good agreement is obtained showing that the time evolution of the symmetric state is well reproduced by an exponential decay following . In the next step we pick a set of randomly drawn vibrational frequencies ν_1_,...ν_*n*_max__ in the window 0–4Ω and Huang–Rhys factors *s*_1_,...*s*_*n*_max__ in the interval [0,0.2] (with an average *s* = 0.1). The results plotted in Figure 4a show that a good fit is obtained with the simplified result of eq 1 which assumes evenly spaced vibrational frequencies and a Huang–Rhys factor at the level of the distribution average *s* = 0.1. Furthermore, an average of the numerical results over 200 random realizations predicts excellent agreement to the linear fit predicted by eq 1.

As aggregates are immersed in solvents and usually under room temperature conditions, they are expected to present a large inhomogeneous broadening at the level of THz. Moreover, monomers are not placed in an exactly equidistant chain, resulting in further shifts in molecular energies. We consider a distribution of the  monomer frequencies around ω_0_ such that the frequency of each monomer becomes ω_0_ + δ_*j*_ where δ_*j*_ is randomly drawn from a distribution of width δω. On a collective basis, the symmetric state couples to any asymmetric state and acquires a shift as well.^21^ The coupling of  to a state *q* mediated by disorder is simply given by the Fourier transform of the distribution , while the shift of the collective state  is the average of the distribution, thus close to zero. According to ref (21), disorder-induced couplings introduce an additional loss channel, thus slightly increasing the Kasha rate. This is indeed consistent with numerical simulations shown in Figure 4f where the dynamics of the symmetric state without disorder and with considerable disorder at δω = Ω and δω = 2Ω are compared. The result is that the analytically derived loss rate holds well even for considerable disorder levels.

Finally, let us provide some estimates of the expected time scale for such a Kasha process in the aqueous dye molecule CV, Rhodamine 800, and BChla. The results are listed in Table 1 with parameters estimated from refs 15–18. The upshot is that analytically estimated values for the Kasha time scale run in the tens of femtoseconds.

**Table 1:** Estimates of the Time Scale  (Femtoseconds) of the Kasha Process According to Eq 1^a^

dye molecule	*d* (nm)	| 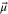 | (D)	γ_0_ (MHz)	*n*_max_	*s*	κ_S_^–1^ (fs)
CV	∼2	1.8	455	12	0.09	13
rhodamine 800	∼2	1.8	525	16	0.067	30
BChl a	∼1	6.4	335	25	0.07	34

a The specific dyes considered are CV with Ω_0_ ≈ 23 THz,^15,16^ rhodamine 800 with Ω_0_ ≈ 8 THz,^17^ and BChla with Ω_0_ ≈ 6 THz, measured at room temperature.^18^

## Discussion and Conclusions

We investigated the time dynamics following the photoexcitation of molecular aggregates in the side-by-side configuration. These are a perfect showcase of cooperative phenomena as their photophysics is naturally characterized by coherent and incoherent effects brought on by the positioning of individual monomers in the near field of each other. Effects widely explored in quantum optics, such as Dicke superradiance and subradiance, naturally occur in the theoretical description of such compounds, albeit in the presence of more complex, additional interactions between electrons and a vast number of molecular vibrations. We have provided a theoretical approach to the dynamics of collective electronic states making the connection, at the analytical and numerical level, with the physical mechanism introduced long ago by Kasha, stating that photon emission (fluorescence or phosphorescence) occurs in appreciable yield only from the lowest excited state of a given multiplicity. Our analytical conclusions predict that the Kasha loss rate from the symmetric, high energy, optically addressable state to the lower energy states of an H-aggregate is roughly independent of the number of monomers but strongly dependent on the number of low-energy vibrational modes which can be excited and then dissipate the accumulated energy afterward. Further investigations will focus on aspects, such as the role of quantum coherence in such systems. An important direction is the application of the methods presented in this manuscript to photosynthetic systems under various conditions of illumination, ranging from spatially and time coherent laser light to spatially and time incoherent light sources.

## Supporting Information Available

The following files are available free of charge. The Supporting Information is available free of charge at https://pubs.acs.org/doi/10.1021/acs.jpca.4c00342.

Analytical derivation of the results in the main text and description of the quantum optical formalism (PDF)
