# Supplementary material for: Scaling Law for Kasha’s Rule in Photoexcited Molecular Aggregates
Source: J Phys Chem A. 2024 Apr 3;128(19):3910–5. doi: 10.1021/acs.jpca.4c00342 (PMC11103697; doi:10.1021/acs.jpca.4c00342)
Supplement: Supplementary file 1 — jp4c00342_si_001.pdf [file jp4c00342_si_001.pdf]

Supporting Information for Scaling Law for Kasha's Rule in Photoexcited Molecular Aggregates

Raphael Holzinger,[†] Nico S. Bassler,[‡] Helmut Ritsch,[¶] and Claudiu Genes[*,§]

†*Institute for Theoretical Physics, Technikerstraße 21a, 6020 Innsbruck University, Austria, E-mail: raphael.holzinger@uibk.ac.at, Phone: +43 512 507 52287*

‡*Max Planck Institute for the Science of Light, Staudtstraße 2, D-91058 Erlangen, Germany, E-mail: nico.bassler@mpl.mpg.de, Phone: +49 9131 7133632*

¶*Institute for Theoretical Physics, Technikerstraße 21a, 6020 Innsbruck University, Austria, E-mail: helmut.ritsch@uibk.ac.at, Phone: +43 512 507 52213*

§*Max Planck Institute for the Science of Light, Staudtstraße 2, D-91058 Erlangen, Germany, E-mail: caudiu.genes@mpl.mpg.de, Phone: +49 9131 7133622*

E-mail: claudiu.genes@mpl.mpg.de

Phone: +49 9131 7133622

S1. Vibronic coupling

Let us justify the form of the Holstein Hamiltonian in Eq. 2 by following a first-principle derivation for a single nuclear coordinate R of effective mass μ. We assume that, along the nuclear coordinate, the equilibria for ground (coordinate R_g , state vector $|g\rangle$) and excited (coordinate R_e and state vector $|e\rangle$) electronic orbitals are different. Assuming equilibrium positions R_g and R_e for the potential surfaces of electronic ground and excited states, one can

write the total molecular Hamiltonian describing both electronic and vibrational dynamics as

$$\mathcal{H}_{\text{mol}} = \left[\omega_0 + \frac{\hat{P}^2}{2\mu} + \frac{1}{2}\mu\nu^2\left(\hat{R} - R_e\right)^2\right]\sigma^\dagger\sigma + \left[\frac{\hat{P}^2}{2\mu} + \frac{1}{2}\mu\nu^2\left(\hat{R} - R_g\right)^2\right]\sigma\sigma^\dagger, \tag{S.1}$$

where μ is the reduced mass of the vibrational mode and $\sigma = |g\rangle\langle e|$. The kinetic and potential energies are written in terms of the position $\hat{Q}$ and momentum operator $\hat{P}$ describing the nuclear coordinate under consideration, with commutation $[\hat{Q}, \hat{P}] = i$. Introducing oscillations around the equilibria $\hat{Q} = \hat{R} - R_{\text{g}}$ and subsequently $\hat{R} - R_{\text{e}} = \hat{Q} + R_{\text{g}} - R_{\text{e}} =: \hat{Q} - R_{\text{ge}}$ we obtain

$$\mathcal{H}_{\text{mol}} = \frac{\hat{P}^2}{2\mu} + \frac{1}{2}\mu\nu^2\hat{Q}^2 + \omega_0\sigma^\dagger\sigma - \mu\nu^2\hat{Q}R_{\text{ge}}\sigma^\dagger\sigma + \frac{1}{2}\mu\nu^2R_{\text{ge}}^2\sigma^\dagger\sigma. \tag{S.2}$$

We can now rewrite the momentum and position operators in terms of bosonic operators $\hat{Q} = q_{\text{zpm}}(b^\dagger + b)$, $\hat{P} = ip_{\text{zpm}}(b^\dagger - b)$. The bosonic operators satisfy the usual commutation relation $[b, b^\dagger] = 1$ and the zero-point motion displacement and momentum are defined as $q_{\text{zpm}} = 1/\sqrt{2\mu\nu}$ and $p_{\text{zpm}} = \sqrt{\mu\nu/2}$. Reexpressing the terms above yields the Holstein Hamiltonian

$$\mathcal{H}_{\text{mol}} = (\omega_0 + s\nu)\sigma^\dagger\sigma + \nu b^\dagger b - \sqrt{s}\nu(b^\dagger + b)\sigma^\dagger\sigma. \tag{S.3}$$

The dimensionless vibronic coupling strength s is given by $\sqrt{s} = \mu\nu R_{\text{ge}}q_{\text{zpm}}$ (s is called the Huang-Rhys factor and is typically on the order of $\sim 0.01 - 1$).

S2. Radiative and vibrational loss

In a master equation formulation for the system density operator ρ written as $\partial_t\rho = i[\rho, \mathcal{H}] + \mathcal{L}[\rho]$ loss can be included via the Lindblad superoperator

$$\mathcal{L}_\gamma[\rho] = \gamma_{\mathcal{O}}/2\left[2\mathcal{O}\rho(t)\mathcal{O}^\dagger - \mathcal{O}^\dagger\mathcal{O}\rho(t) - \rho(t)\mathcal{O}^\dagger\mathcal{O}\right], \tag{S.4}$$

describing decay at generic rate $\gamma_{\mathcal{O}}$ through a single channel with a generic collapse operator $\mathcal{O}$. For vibrational loss, the collapse rate for each mode m is Γ_m and the corresponding collapse operator is $b_{jm} - \sqrt{s_m}\sigma_j^\dagger\sigma_j$. This form for the collapse operator is derived in analogy to the dissipative physics of optomechanical systems in the ultrastrong coupling regime.[1] The radiative loss is not in diagonal Lindblad form but achieves the following expression $\mathcal{L}_e[\rho] = \sum_{j,j'} \gamma_{jj'}/2 \left[2\sigma_j\rho\sigma_{j'}^\dagger - \sigma_j^\dagger\sigma_{j'}\rho - \rho\sigma_j^\dagger\sigma_{j'}\right]$. This form can be diagonalized and it shows the emergence of $\mathcal{N}$ independent decay channels, each corresponding to some collective electronic superposition state.[2] At very small separation, deep into the subwavelength regime, the fully symmetric superposition decays at a superradiant rate roughly equal to $\mathcal{N}\gamma_0$ while all other states have vanishingly small decay rates (which we will assume in the following to be exactly zero). This is by no means a limitation of our treatment as one can easily generalize this to the case of non-zero decay rates of the dark manifold.[2,3]

S3. Hamiltonian in the collective basis

The Hamiltonian coupling the symmetric state to the dark state manifold is then given by

$$\mathcal{H}_{\text{int}}^{\mathcal{S}\mathcal{A}} = -\sum_{m=1}^{n}\sum_{q\neq 0} \frac{\sqrt{s_m}\nu_m}{\sqrt{\mathcal{N}}}\left(Q_q^{(m)}\mathcal{S}^\dagger\mathcal{A}_q + \text{h.c.}\right), \tag{S.5}$$

via collective vibrations. This coupling is responsible for funneling population into the long lived dark state manifold after the initial driving of the fully symmetric state under uniform illumination. This mechanism is fundamental to understand the dynamics associated with Kasha's rule which we tackle in the main text. In addition, within the dark state manifold an all-to-all coupling Hamiltonian acts with the following form

$$\mathcal{H}_{\text{int}}^{\mathcal{A}\mathcal{A}} = -\sum_{m=1}^{n}\sum_{q\neq q'} \frac{\sqrt{s_m}\nu_m}{\sqrt{\mathcal{N}}}\left(Q_{q-q'}^{(m)}\mathcal{A}_q^\dagger\mathcal{A}_{q'} + \text{h.c.}\right), \tag{S.6}$$

and the sum implies that $q, q' \neq 0$. This indicates that a redistribution of energy takes place within the whole manifold of dark states. After transforming the system Hamiltonian, the energies of the collective states are shifted by the contribution of the symmetric vibrational mode $-\sum_m \sqrt{s_m}\nu_m Q_0^{(m)}/\sqrt{\mathcal{N}}$. The energy shifts can be removed by the collective polaron transformation $U = \prod_q \prod_{m=1}^n e^{i\sqrt{s_m}/\sqrt{\mathcal{N}} P_0^{(m)} \mathcal{A}_q^\dagger \mathcal{A}_q}$ which leads to a renormalization of the collective state energies as $\bar{\omega}_q = \omega_0 + \sum_m s_m\nu_m/2 + \Omega_q$.

S4. Vacuum mediated dipole-dipole coupling rates

The vacuum mediated dipole-dipole interactions for an electronic transition at wavelength λ_0 (corresponding wave vector $k_0 = 2\pi/\lambda_0$) between identical pairs of emitters separated by r_{ij} is given in terms of the free-space electromagnetic Green's tensor $\mathbf{G}(\vec{r}_i - \vec{r}_j, \omega_0) \equiv \mathbf{G}(\vec{r}_{ij}, \omega_0)$, with $\vec{r}_{ij} = \vec{r}_i - \vec{r}_j$, which reads

$$\mathbf{G}(\vec{r}, \omega_0) = \frac{e^{ik_0 r}}{4\pi k_0^2 r^3}\left[(k_0^2 r^2 + ik_0 r - 1)I + (-k_0^2 r^2 - 3ik_0 r + 3)\frac{\vec{r}\otimes\vec{r}}{r^2}\right], \tag{S.7}$$

where $r = |\vec{r}|$. The Green's function $\mathbf{G}_{\alpha\beta}$ is a tensor quantity, with $\{\alpha, \beta\} = \{x, y, z\}$ which is determined by the polarization direction of the dipoles. In order to obtain the dipole-dipole couplings for H-aggregates we chose linear polarization in z-direction for all molecules, namely we take $\vec{\mu}_z^* \cdot \mathbf{G} \cdot \vec{\mu}_z$.

S5. Deriving rate equations

Starting from the Holstein Hamiltonian in Eqs. (5)-(6) for $\mathcal{N}$ identical molecules with n vibrational modes each. The Heisenberg equations for the collective electronic modes are given by

$$\dot{\mathcal{S}} = -i\Big(\Omega_{\mathcal{S}} - \frac{\gamma_{\mathcal{S}}}{2}\Big)\mathcal{S} + \frac{i\sqrt{s}_m\nu_m}{\sqrt{\mathcal{N}}}\sum_{m=1}^{n}\sum_{q} Q_q^{(m)}\mathcal{A}_q + \text{noise}, \tag{S.8a}$$

$$\dot{\mathcal{A}}_q = -i\Omega_q\mathcal{A}_q + \sum_{m=1}^{n}\frac{i\sqrt{s_m}\nu_m}{\sqrt{\mathcal{N}}}\Big(Q_q^{(m)\dagger}\mathcal{S} + \sum_{q'\neq q} Q_{q-q'}^{(m)}\mathcal{A}_{q'}\Big) + \text{noise}. \tag{S.8b}$$

The collective noise terms will be neglected from now on as they do not contribute to the transfer process.

To calculate the transfer rate from the symmetric state to the antisymmetric states we assume some initial population in the symmetric state and no population in the antisymmetric states, additionally we assume that the symmetric state decays independently and formally integrate

$$\mathcal{S}(t) = S(0)e^{-(i\Omega_{\mathcal{S}}+\gamma_{\mathcal{S}}/2)t}, \tag{S.9a}$$

$$\mathcal{A}_q(t) = A_q(0)e^{-i\Omega_q t} + \sum_{m=1}^{n}\frac{i\sqrt{s_m}\nu_m}{\sqrt{\mathcal{N}}}\int_0^t dt' e^{-i\Omega_q(t-t')}\Big(Q_q^{(m)}(t')\mathcal{S}(t') + \sum_{q'\neq q} Q_{q-q'}^{(m)}(t')\mathcal{A}_{q'}(t')\Big), \tag{S.9b}$$

and for the expectation value of the populations we get

$$\dot{\langle\mathcal{S}^\dagger\mathcal{S}\rangle} = -\gamma_{\mathcal{S}}\langle\mathcal{S}^\dagger\mathcal{S}\rangle - \sum_{m=1}^{n}\sum_{q}\frac{2\sqrt{s_m}\nu_m}{\sqrt{\mathcal{N}}}\,\text{Im}\langle\mathcal{S}^\dagger\mathcal{A}_q Q_q^{(m)}\rangle, \tag{S.10a}$$

$$\dot{\langle\mathcal{A}_q^\dagger\mathcal{A}_q\rangle} = -\sum_{m=1}^{n}\frac{2\sqrt{s_m}\nu_m}{\sqrt{\mathcal{N}}}\,\text{Im}\Big(\langle\mathcal{A}_q^\dagger\mathcal{S}Q_q^{(m)}\rangle + \sum_{q'\neq q}\langle\mathcal{A}_q^\dagger\mathcal{A}_{q'}Q_{q'-q}^{(m)}\rangle\Big). \tag{S.10b}$$

Therefore the terms $-2\sqrt{s}_m\nu_m/\sqrt{\mathcal{N}}\,\text{Im}\langle\mathcal{A}_q^\dagger\mathcal{S}Q_q^{(m)}\rangle$ will be responsible for population transfer from the symmetric to the antisymmetric state with quasi-momentum q at a rate $\kappa_{\mathcal{S}\to q}^{(m)}$. We can calculate the rates explicitly up to order $\mathcal{O}(s_m\nu_m^2)$ and assuming that correlations between vibronic and electronic operators factorize.

$$-2\sqrt{s}_m\nu_m/\sqrt{\mathcal{N}}\langle\mathcal{A}_q^\dagger\mathcal{S}Q_q^{(m)}\rangle = -is_m\nu_m^2\int_0^t dt' e^{-\Omega_q(t-t')}\langle Q_q^{(m)}(t')Q_q^{(m)}(t)\rangle\langle\mathcal{S}^\dagger(0)\mathcal{S}(0)\rangle e^{-\epsilon_S t'}e^{-\epsilon_S^* t}$$

$$= -is_m\nu_m^2\langle\mathcal{S}^\dagger(0)\mathcal{S}(0)\rangle\frac{e^{-\gamma_S t} - e^{-((\Gamma_m+\gamma_S)/2+i(\Omega_S-\Omega_q-\nu_m))t}}{(\Gamma_m+\gamma_S)/2 + i(\Omega_S-\Omega_q-\nu_m)}, \quad \text{(S.11)}$$

where we defined $\epsilon_S = -(\gamma_S/2 - i\Omega_S)$ and used the fact that different vibrational modes are uncorrelated at all times, i.e. $\langle Q_q^{(m')}(t')Q_q^{(m)}(t)\rangle = 0$ for $m' \neq m$. The correlations for $Q_q^{(m)}$ are evaluated assuming free evolution of the vibrations (to lowest order) and zero temperature for the vibrational modes:

$$\langle Q_q^{(m)}(t')Q_q^{(m)}(t)\rangle = \frac{1}{\mathcal{N}}\sum_{j=1}^{\mathcal{N}}\langle b_{jm}(t')b_{jm}^\dagger(t)\rangle = e^{-(\Gamma_m/2-i\nu_m)(t-t')}. \quad \text{(S.12)}$$

The transfer rate can be written as

$$\kappa_{\mathcal{S}\to q}^{(m)} = \frac{2s_m\nu_m^2(\Gamma_m+\gamma_{\mathcal{S}})/\mathcal{N}}{(\Gamma_m+\gamma_{\mathcal{S}})^2 + 4(\Omega_{\mathcal{S}}-\Omega_q-\nu_m)^2}, \quad \text{(S.13)}$$

given fast vibrational relaxation rates $\Gamma_m \gg \gamma_S$ compared to the electronic decay rates.

S6. Single excitation subspace

The numerical diagonalization and subsequent time dynamics are evaluated in the single-excitation sector for both the electronic and vibrational degrees of freedom. This allows to rewrite the effective Hamiltonian in non-hermitian form as ($\hbar = 1$)

$$\mathcal{H}_{\text{eff}} = \sum_{j=1}^{\mathcal{N}}\Big(h^{(j)} + \sum_{j'=1}^{\mathcal{N}}\Big(\Omega_{jj'} - i\frac{\gamma_{jj'}}{2}\Big)\sigma_j^\dagger\sigma_{j'} - \frac{i}{2}\sum_{m=1}^{n}\Gamma_m\mathcal{O}_{jm}^\dagger\mathcal{O}_{jm}\Big), \quad \text{(S.14)}$$

where $h^{(j)}$ is defined in Eq. 1 and $\mathcal{O}_{jm} = b_{jm} - \sqrt{s_m}\sigma_j^\dagger\sigma_j$. The dynamics of the electron-vibron density matrix ρ can be described by a von Neumann equation of the form

$$i\frac{\mathrm{d}}{\mathrm{dt}}\rho(t) = [\mathcal{H}_{\text{eff}}\rho - \rho\mathcal{H}_{\text{eff}}], \tag{S.15}$$

and the expectation value of observable $\mathcal{O}$ becomes $\bar{\mathcal{O}} = \mathrm{tr}(\rho\mathcal{O})$. However, instead of solving the von Neumann equation directly, one can use the quantum jump formalism to evaluate single stochastic quantum trajectories using the Monte Carlo wave function method (MCWF). For large numbers of trajectories, the statistical average then approximates the result of the Master equation. The huge advantage is that instead of describing the state of the quantum system by a density matrix of size $\mathcal{N}^4 \times n^2$ these trajectories work in terms of state vectors of size $\mathcal{N}^2 \times n$. This is somewhat counteracted by the stochastic nature of the formalism which makes it necessary to repeat the simulation until the wanted accuracy is reached. It turns out, however, that for many cases, especially for high dimensional quantum systems, the necessary number of repetitions is much smaller than the system size $\mathcal{N}^2 \times n$ and therefore using the MCWF method is advantageous.

The system size stems from the fact, that in the single excitation subspace for both electronic and vibronic modes a general state vector can be written as

$$|\Psi\rangle = \sum_{j=1}^{\mathcal{N}} \alpha_{jj'}^{(m)} |g, g, ...e_j, ...\rangle \otimes \sum_{m=1}^{n}\sum_{j'=1}^{\mathcal{N}} |0, 0, ...1_{j'}, ...\rangle^{(m)}, \tag{S.16}$$

with coefficients $\alpha_{jj'}^{(m)}$ and where the first part refers to the electronic excitation of molecule j and the second part to the excitation of the m-th vibrational mode of molecule j'. Thus, the single excitation assumption substantially reduces the Hilbert space dimension from $2^{\mathcal{N}} \times n_{\text{cut}}^{\mathcal{N}} \times n$ to $\mathcal{N}^2 \times n$ (where n_{cut} is the cut-off of the Fock space dimension for the vibrational modes), allowing the simulation of mesoscopic numbers of molecules.

References

(1) Hu, D.; Huang, S.-Y.; Liao, J.-Q.; Tian, L.; Goan, H.-S. Quantum coherence in ultrastrong optomechanics. *Phys. Rev. A* **2015**, *91*, 013812.

(2) Reitz, M.; Sommer, C.; Genes, C. Cooperative Quantum Phenomena in Light-Matter Platforms. *PRX Quantum* **2022**, *3*, 010201.

(3) Holzinger, R.; Oh, S. A.; Reitz, M.; Ritsch, H.; Genes, C. Cooperative subwavelength molecular quantum emitter arrays. *Phys. Rev. Res.* **2022**, *4*, 033116.
